# Predictors of mortality in COVID-19 patients at Kinshasa Medical Center and a survival analysis: a retrospective cohort study

**DOI:** 10.1186/s12879-021-06984-x

**Published:** 2021-12-20

**Authors:** Yannick Nlandu, Danny Mafuta, Junior Sakaji, Melinda Brecknell, Yannick Engole, Jessy Abatha, Jean-Robert Nkumu, Aliocha Nkodila, Marie-France Mboliassa, Olivier Tuyinama, Dauphin Bena, Yves Mboloko, Patrick Kobo, Patrick Boloko, Joseph Tshangu, Philippe Azika, Jean-Pierre Kanku, Pally Mafuta, Magloire Atantama, Jean-Michel Mavungu, Rosita Kitenge, Asma Sehli, Karel Van Eckout, Cathy Mukuku, Léo Bergeret, David Benchetritt, Golan Kalifa, Ahmed Rodolphe, Justine Bukabau

**Affiliations:** 1 Intensive Care Unit, Kinshasa Medical Center, Kinshasa, Democratic Republic of the Congo; 2 Nephrology Unit, Kinshasa University Hospital, Kinshasa, Democratic Republic of the Congo; 3 Faculty of Public Health, Lomo University, Kinshasa, Democratic Republic of the Congo

**Keywords:** COVID-19, Mortality, African people, Procalcitonin, Acute kidney injury

## Abstract

**Background:**

Despite it being a global pandemic, there is little research examining the clinical features of severe COVID-19 in sub-Saharan Africa. This study aims to identify predictors of mortality in COVID-19 patients at Kinshasa Medical Center (KMC).

**Methods:**

In this retrospective, observational, cohort study carried out at the Kinshasa Medical Center (KMC) between March 10, 2020 and July 10, 2020, we included all adult inpatients (≥ 18 years old) with a positive COVID-19 PCR result. The end point of the study was survival. The study population was dichotomized into survivors and non-survivors group. Kaplan–Meier plot was used for survival analyses. The Log-Rank test was employed to compare the survival curves. Predictors of mortality were identified by Cox regression models. The significance level of p value was set at 0.05.

**Results:**

432 patients with confirmed COVID-19 were identified and only 106 (24.5%) patients with moderate, severe or critical illness (mean age 55.6 ± 13.2 years old, 80.2% were male) were included in this study, of whom 34 (32%) died during their hospitalisation. The main complications of the patients included ARDS in 59/66 (89.4%) patients, coagulopathy in 35/93 (37.6%) patients, acute cardiac injury in 24/98 (24.5%) patients, AKI in 15/74 (20.3%) patients and secondary infection in 12/81 (14.8%) patients. The independent predictors of mortality were found to be age [aHR 1.38; 95% CI 1.10–1.82], AKI stage 3 [aHR 2.51; 95% CI 1.33–6.80], proteinuria [aHR 2.60; 95% CI 1.40–6.42], respiratory rate [aHR 1.42; 95% CI 1.09–1.92] and procalcitonin [aHR 1.08; 95% CI 1.03–1.14]. The median survival time of the entire group was 12 days. The cumulative survival rate of COVID-19 patients was 86.9%, 65.0% and 19.9% respectively at 5, 10 and 20 days*.* Levels of creatinine (p = 0.012), were clearly elevated in non-survivors compared with survivors throughout the clinical course and increased deterioration.

**Conclusion:**

Mortality rate of COVID-19 patients is high, particularly in intubated patients and is associated with age, respiratory rate, proteinuria, procalcitonin and acute kidney injury.

## Background

In December 2019, the first case of coronavirus disease 2019 (COVID-19), caused by the severe acute respiratory syndrome coronavirus 2 (SARS-COV-2) was detected in China [1]. Three months later, due to its rapid global spread, the World Health Organization (WHO) declared the outbreak a pandemic [1]. By the end of September 2020, the WHO had reported that SARS-COV-2 had infected at least 32.7 million people and was responsible for the deaths of more than one million [2]. The clinical manifestations of this new disease vary widely in severity; ranging from no or mild symptoms to patients with pneumonia progressing rapidly to acute respiratory distress syndrome (ARDS), multi-organ failure and death [3]. There is not only a huge disparity in the severity of this disease but also in its impact globally. From the onset of the pandemic, WHO predicted high morbidity and mortality rates in African countries. However, this has not transpired, with Africa reporting much lower rates than most of the rest of the world [4–6]. Notwithstanding the huge amount of global research investigating these morbidity and mortality disparities, most of these data emanate from non-African countries.

The Democratic Republic of Congo (DRC) situated in Central Africa, is a vast country with a surface area equivalent to Western Europe with an population of 89,561,403 [4]. The country reported its first case of COVID-19 on March 10, 2020. This was an imported case from France [7]. As of September 27, 2020, there have been a total of 10, 592 positive cases, with a mortality of 271 [2]. To date there is a paucity of research on COVID-19 from the DRC, two papers have been identified. The objective of this study was to identify the factors/determinants for COVID-19 related mortality by comparing the demographic and clinical characteristics of patients diagnosed with COVID-19 admitted to the Kinshasa Medical Center (KMC), located in Kinshasa, the capital of the Democratic Republic of Congo (DRC).

## Methods

### Study design, setting and population

This retrospective, observational, cohort study was carried out at the KMC, a private hospital officially designated for the treatment of COVID-19 between March 10, 2020 and July 10, 2020. The sampling for this study was consecutive. The inclusion criteria were strictly based on laboratory confirmation of SARS-CoV-2 by qualitative reverse-transcriptase polymerase chain reaction (RT-PCR) assay of nasopharyngeal swabs. Only patients who demonstrated signs of moderate, severe and critical illness were admitted to hospital. There was no formal determination of sample size and all patients meeting the inclusion criteria were recruited.

### Ethics approval

This study was carried out in strict compliance with the recommendations of the Declaration of Helsinki III [8]. The data were collected anonymously and confidentially. The information obtained during the history and clinical examination was transcribed into pre-established and pre-coded investigation sheets while respecting the confidentiality and privacy of patients. Our research projects on Covid-19 had been authorized by the National Ethics committee of Health, Democratic Republic of Congo (N°225/CNES/BN/PMMF/2020). The need for ethics approval and consent to participate were waived by the National Ethics committee of Health, Democratic Republic of Congo because of the urgency and unprecedented nature of the COVID-19 pandemic. Administrative permissions to access the raw data were granted by the Kinshasa Medical Center (KMC) direction.

### Data collection

Clinical data were extracted manually from the KMC electronic patient database. Information about demographic characteristics (age and gender); the existence of any chronic conditions (hypertension, diabetes mellitus, chronic kidney disease);initial symptoms (fever, cough, shortness of breath, chills, dyspnea, fatigue, nausea, vomiting, and diarrhea); vital signs (temperature, respiratory rate [RR], heart rate [HR], and blood oxygen saturation [BOS]); and laboratory tests (haemoglobin [Hb], white blood cells [WBC], neutrophils, lymphocytes, platelets, albumin, creatinine, urea, lactate dehydrogenase [LDH], creatine kinase [CK], D-dimer, C-reactive protein [CRP], procalcitonin [PCT], fibrinogen, high sensitivity Troponin I [hsTNI], electrolytes) and thoracic computerized tomography scan (CT) score were all collected from the time of the admission except for CRP, procalcitonin and creatinine which were repeated systematically on day 1, 3 and 7 in according to the hospital protocol**.** In addition, we collected information about the treatment received (administration of antibiotics, corticosteroids, oxygen therapy, mechanical ventilation or haemodialysis, complications, and outcomes during the hospital admission. Blood parameters were categorized according to normal reference ranges used in hospital.

### Definitions

Fever was defined as axillary temperature of at least 37·3 °C. Hypertension was recorded if the patient was taking any antihypertensive drug or had two separate BP measurements ≥ 140/90 mmHg [9]. Secondary infection was diagnosed when patients showed clinical symptoms or signs of pneumonia or bacteraemia and a positive culture of a new pathogen was obtained from blood samples after admission [10]. The diabetes diagnosis was based on criteria from the American Diabetes Association as a presence of a fasting plasma glucose level of > 126 mg/dL or usage of antidiabetic drug [11]. CKD was defined according to KDIGO definition [12]. ARDS was defined according to the Berlin Definition [13]. Acute Kidney Injury (AKI) was diagnosed according to KDIGO clinical practice guidelines based on the serum creatinine levels [14]. Acute cardiac injury was diagnosed if the serum concentration of HsTNI was above the upper limit of the reference range (> 28 pg/mL) [15]. Coagulopathy was defined as a prothrombin time ratio (PTr) of less than 70% [16].

On admission each patient had a thoracic CT scan that was assessed for severity of pulmonary involvement. A semi-quantitative CT scoring system was calculated based on the extent of lobar involvement (0:0%; 1: < 5%; 2:5–25%; 3:26–50%; 4:51–75%; 5: > 76% [17].

### Statistical analysis

The data was entered and encoded using the Epi info 3.5 software. Data analyzes were performed using SPSS version 21 software. Descriptive statistics consisted of calculating the mean and standard deviation for quantitative data with Gaussian distribution; the median and interquartile range (IQR) for quantitative data with non-Gaussian distribution. Proportions were used for categorical data and percentage are based on the total number of non-missing value.

Pearson’s Chi-square test or Fisher’s exact test was used to compare the proportions. For continuous variables, the comparisons between the survivor and non-survivor groups were made using student’s t-test (variables normally distributed) or Mann Whitney’s test (variables not normally distributed). Kaplan Meier’s method was used to describe the survival between the date of admission in KMC care and death (complete data) and the end of the study (censored data). The Log-rank test was used to compare survival curves. Factors associated with mortality in unadjusted univariable cox regression were included in a multivariable cox regression model to identify independent factors associated with mortality, the Odd ratio (OR) was calculated for each independent variable. We excluded variables from the univariable analysis if their between-group differences were not significant, if the number of events was too small to calculate odds ratios. Only significant variables in univariable cox regression were retained in the final model. A p value < 0.05 was considered the threshold of statistical significance.

## Results

**Fig. 1:**
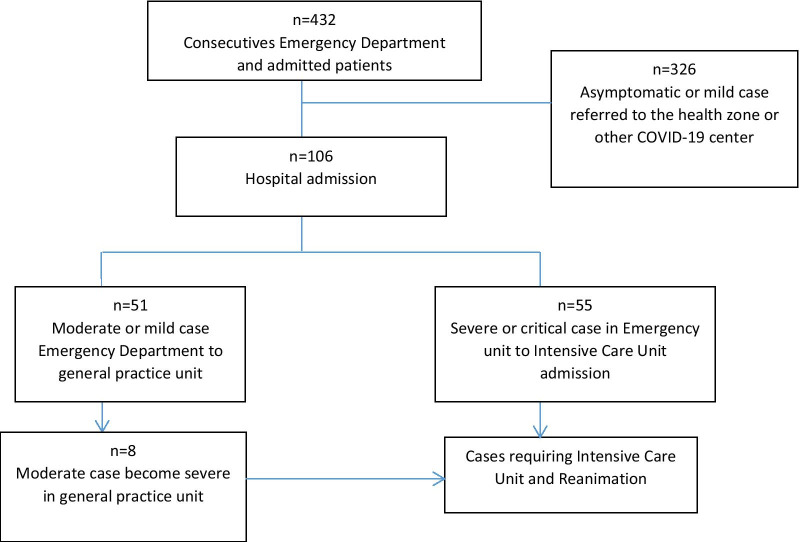
Flow chart of study population selection

**Fig. 2:**
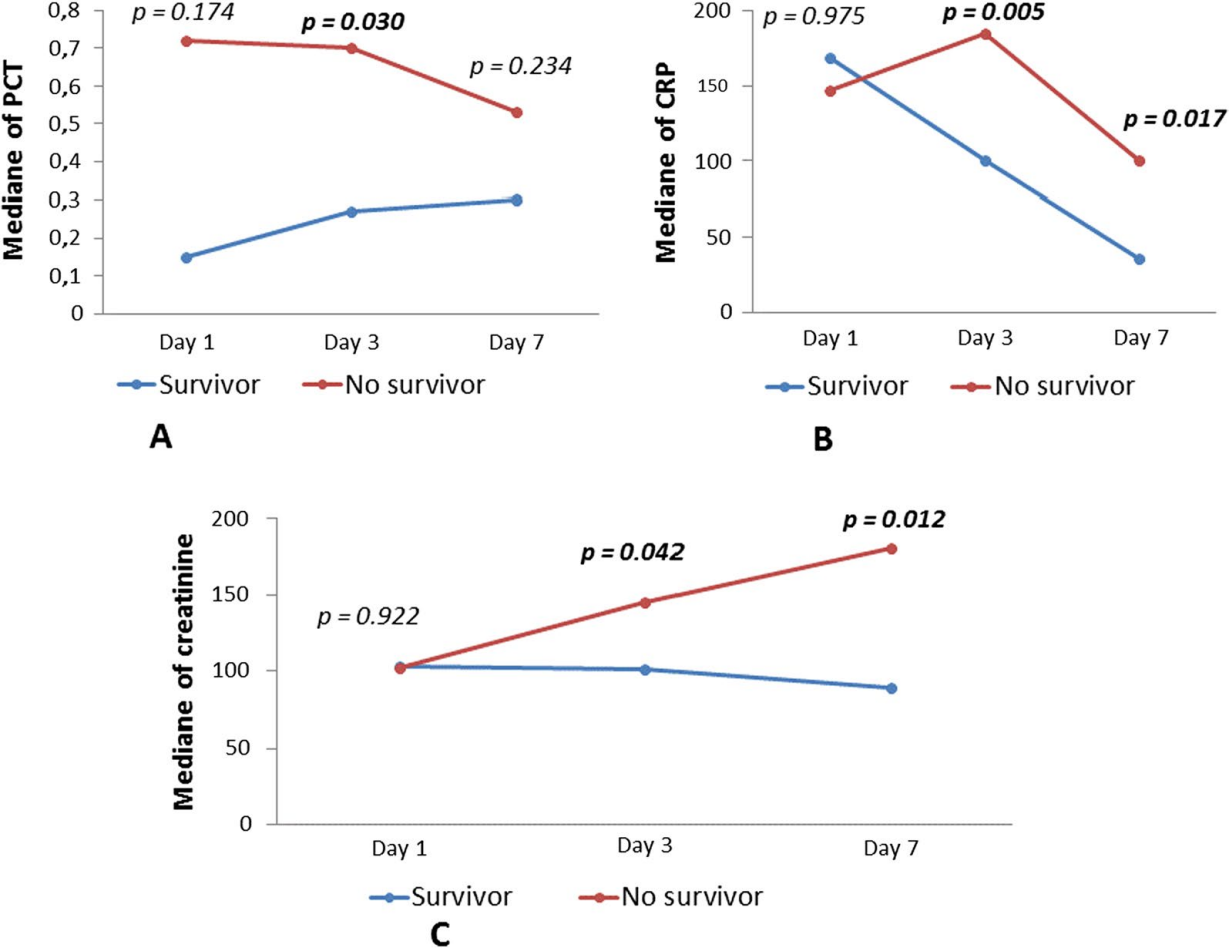
Temporal changes in laboratory markers from illness onset in patients hospitalised with COVID-19 Figure shows temporal changes in Procalcitonin (**A**), C-reactive protein (**B**) and creatinin (**C**). Differences between survivors and non-survivors were significant for all time points shown

**Fig. 3:**
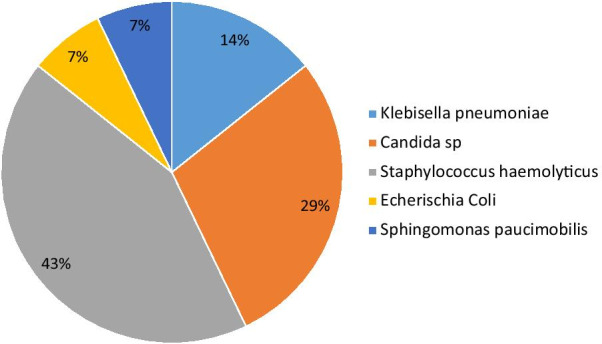
Diagram of main secondary infection in COVID-19 patients

**Fig. 4:**
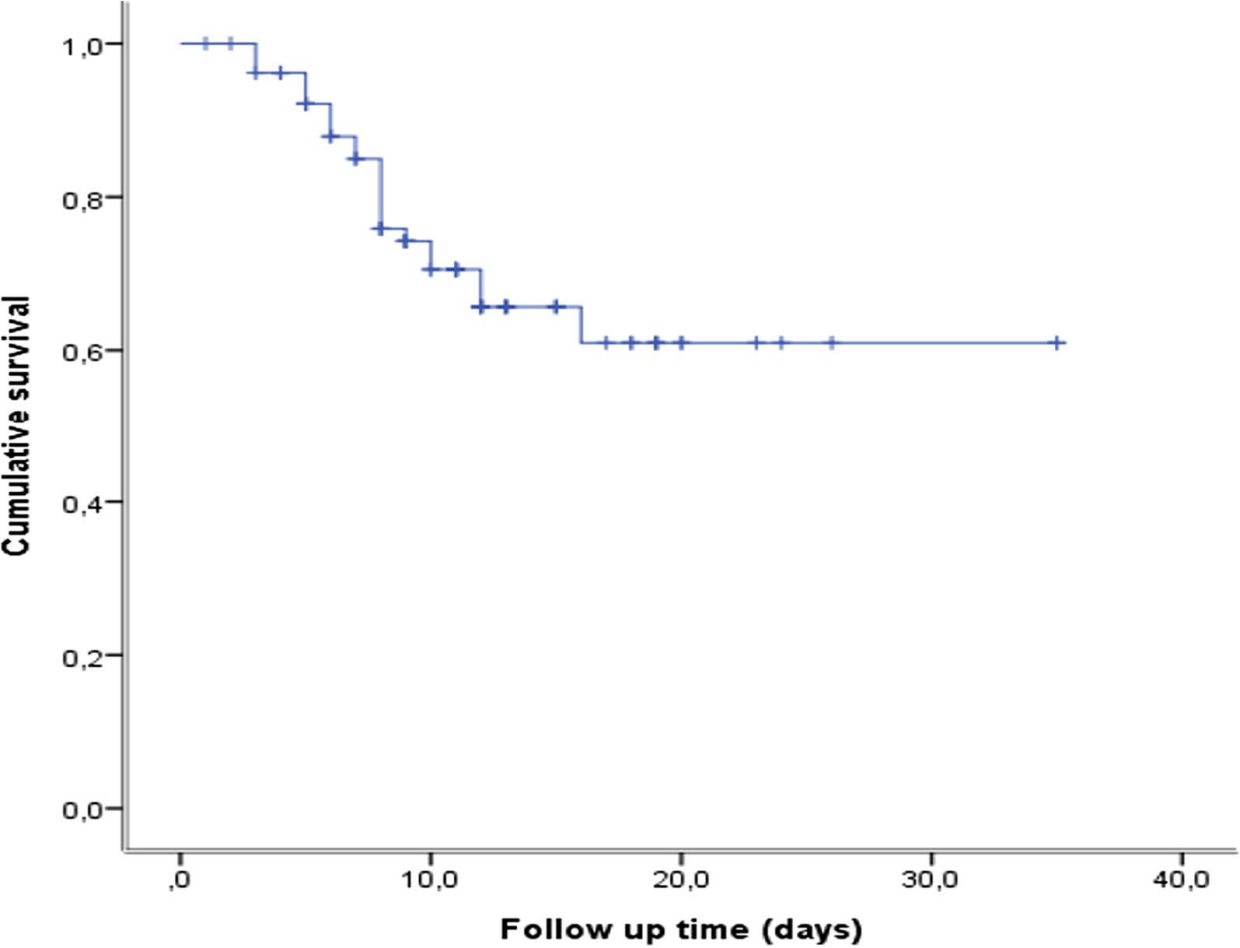
Survival Curve of COVID-19 patients study population

**Fig. 5:**
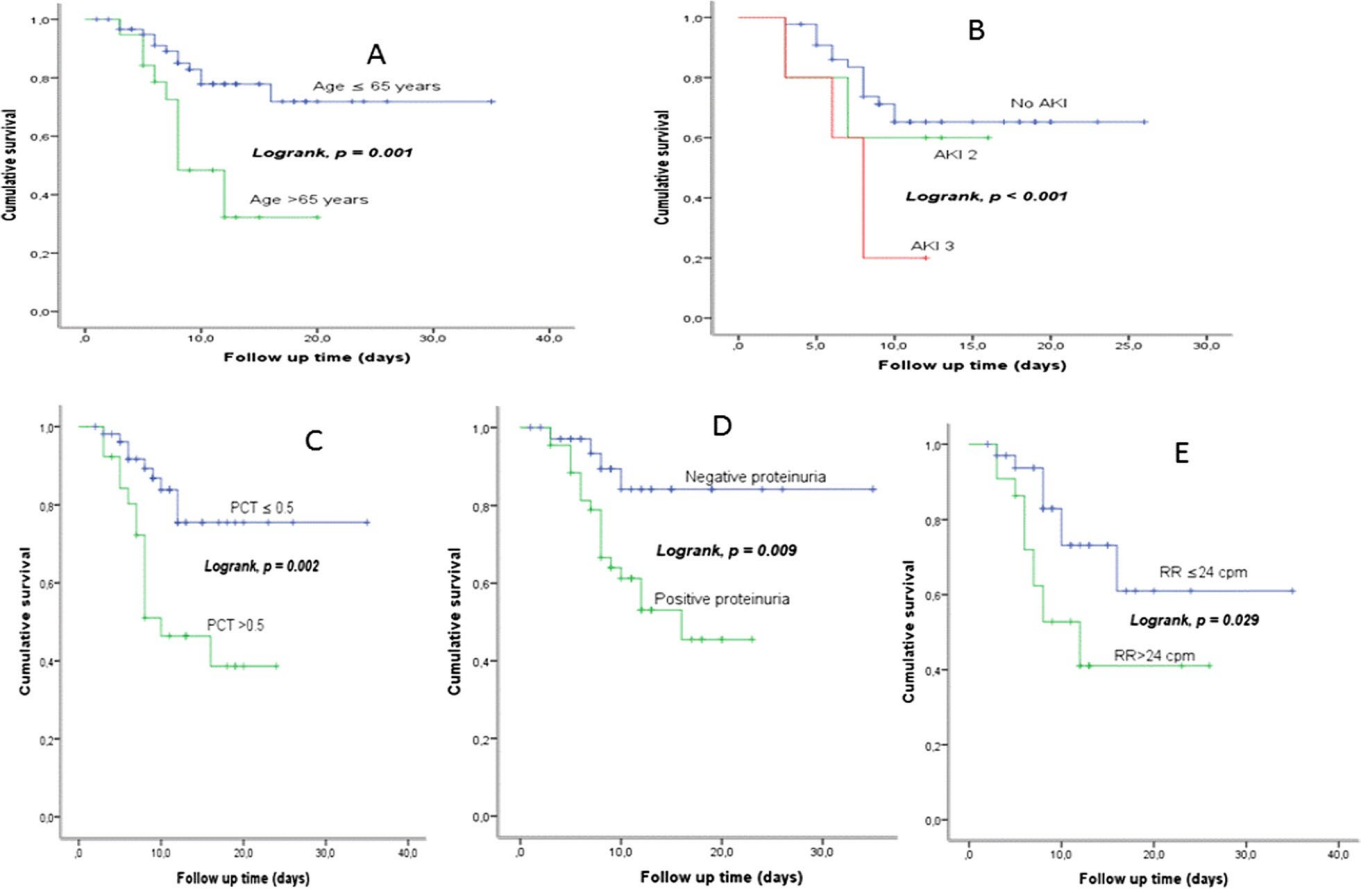
Survival Curves of COVID-19 patients according to Age (**A**), AKI (**B**), procalcitonin (**C**), proteinuria (**D**) and respiratory rate (**E**) status at presentation

Of 432 consecutive patients with COVID-19 who were admitted to the Hospital Emergency Department at KMC between March 10, 2020 and July 10, 2020, only 106 were hospitalized and followed during the study period (Fig. 1). The baseline characteristics of these 106 patients are summarised in Table 1 and their laboratory findings and chest CT scan score in Table 2. 34 patients died during hospitalisation and 72 were discharged. The mean age of the admitted patients was 55.6 ± 13.2 years, including 26 (24.5%) patients over 65 years old. The majority were male (80.2%) with hypertension being the main comorbidity in 62 (58.2%) patients. The median (IQR) time from COVID-19 symptoms onset to hospital admission was 7 (5.8–10.0) days, whereas the median time to death was 22.0 (14.0–33.0). Fever and cough were the most common initial symptoms (65.1% and 55.7%, respectively). On admission, the median axillary temperature was 37.1 °C (IQR: 36.6–38.3 °C). The median respiratory rate was 22/min (IQR: 20–29/min) and median blood oxygen saturation on room air was 89% (IQR: 82–92%). Compared between the two groups, the patients in non-survivors group had significantly higher age (61.3 ± 12 vs 52.9 ± 13), Systolic Blood Pressure (145.5 ± 17.4 vs 137.0 ± 17), Lactate dehydrogenase (604 [244–874.8] vs 362.5 [228.3–551.8]), HDL cholesterol (1.19 [0.75–1.55] vs 0.85 [0.66–1.08]), troponin (20.8 [10.3–90.5] vs 4.9 [2.0–16.9]), procalcitonin (0.360 [0.185–2.583] vs 0.140 [0.06–0.440]) and lower PaO2/FiO2 ratio (67.6 [57.9–96.5] vs 145.5f [73.1–251.2]). The patients in non-survivors group had also significantly more count of neutrophil (5120.5 [3748–7815] vs 3555.7 [2630–5911.5]) (Table 2). The frequency of complications was higher in non-survivors than survivors (Table 3). The main Complications of the patients included ARDS in 59/66 (89.4%) patients, coagulopathy in 35/93 (37.6%) patients, acute cardiac injury in 24/98 (24.5%) patients, AKI in 15/74 (20.3%) patients and secondary infection in 12/81 (14.8%) patients (Table 3). Only 24 patients were on corticosteroids. All 28 (26.4%) patients who required mechanical ventilation (MV) died. The median time from illness onset to invasive mechanical ventilation was 15 days (9.0–22.0). 14 (13.2%) patients received renal replacement therapy. Some laboratory parameters were tracked from illness onset (Fig. 1). Levels of CRP, PCT and creatinine were clearly elevated in non-survivors compared with survivors throughout the clinical course (Fig. 2). As of July 10, 2020, 34 (32.0%) patients had died; of those that died a total of 17.6% (6/34) had secondary infections. The main bacterial infection found were *Staphylococus haemolyticus* (Fig. 3). The median (IQR) length of stay from hospitalization to discharge was 18(15–22) days, while the median (IQR) time from hospitalization to death was 22 (14–33) days. Kaplan Meir survival curve of the study population is illustrated in Fig. 4. The median survival time of the entire group was 12 days. The cumulative survival rate of COVID-19 patients was 86.9%, 65.0% and 19.9% respectively at 5, 10 and 20 days. The Kaplan-Meir curves showed a better survival in younger patients, in patients with No AKI and in patients who have a procalcitonin level below 0.5. Patients with no proteinuria and lower respiratory rate at presentation have also a better survival (Fig. 5). Multivariable analysis (Table 4) showed age [aHR 1.38; 95% CI 1.10–1.82], AKI stage 3 [aHR 2.51; 95% CI 1.33–6.80], proteinuria [aHR 2.60; 95% CI 1.40–6.42], RR [aHR 1.42; 95% CI 1.09–1.92] and procalcitonin [aHR 1.08; 95% CI 1.03–1.14] as factors independently associated with an increased risk of mortality.

**Table 1:** Clinical features at admission

	Total (n = 106)	Non-survivors (n = 34)	Survivors (n = 72)	p-value
Age, years	55.6 ± 13.2	61.3 ± 12.0	52.9 ± 13.0	0.002
> 65 years	26 (24.5)	15 (44.1)	11 (15.3)	0.001
Sex				
Females	21(19.8)	6 (17.6)	15 (20.8)	0.701
Males	85(80.2)	28 (82.4)	57 (79.2)	
Comorbidities				
Hypertension	62 (58.5)	21 (61.8)	41 (56.9)	0.638
Diabetes melitus	35(33.0)	14 (41.2)	21 (29.2)	0.220
CKD	5 (4.7)	2 (5.9)	3 (4.2)	0.697
SBP, mm Hg	139.8 ± 17.5	145.5 ± 17.4	137.0 ± 17.0	0.036
DBP, mm Hg	84.9 ± 13.9	87.0 ± 17.6	83.8 ± 11.7	0.327
HR, bpm	92.1 ± 15.1	93.6 ± 17.5	91.3 ± 14.0	0.513
RR, cycle/min	22.0 (20.0–29.0)	27.5 (22.0–35.0)	20.0 (20.0–26.0)	0.002
RR > 24 cycles/min	26 (36.6)	14 (58.3)	12 (25.5)	0.007
T, °C	37.1 (36.6–38.3)	37.5 (36.6–38.5)	37.0 (36.6–38.0)	0.178
Fever	69 (65.1)	22 (64.7)	47 (65.3)	0.954
Cough	59 (55.7)	22 (64.7)	37 (51.4)	0.198
Dyspnea	42 (39.6)	14 (41.2)	28 (38.9)	0.822
Asthenia	42 (39.6)	11 (32.4)	31 (43.1)	0.293
Symptoms, days	7.0 (5.8–10.0)	7.0 (5.0–10.0)	7.0 (6.0–8.5)	0.682

Data are mean ± standard, median (IQR), n (%), or n/N (%). p values were calculated by Mann–Whitney U test, χ^2^ test, or Fisher’s exact test, as appropriate. *Bpm* beats per minutes, *CKD* chronic kidney disease, *DBP* diastolic blood pressure, *HR* heart rate, *RR* respiratory rate. χ^2^ test comparing all subcategories

**Table 2:** Biological and radiological characteristics at admission

Variable	Total (n = 106)	Non-survivors (n = 34)	Survivors (n = 72)	p-value
Glycemia, mg/dl	124.0 (103.0–176.0)	136.5 (100.5–180.8)	119.0 (103.5–171.0)	0.614
WBC count, × 10^3^/l	6.2 (4.8–8.7)	7.0 (5.3–9.96)	5.9 (4.5–8.1)	0.056
< 4.0	10 (11.0)	2 (7.4)	8 (12.5)	0.617
4.0–10.0	80 (87.9)	25 (92.6)	55 (85.9)	
> 10.0	1 (1.1)	0 (0)	1 (1.6)	
Neutrophils count, × 10^3^/l	4.3 (2.92–6.38)	5.12 (3.75–7.82)	3.56 (2.63–5.91)	0.021
Lymphocytes count, × 10^3^/l	1.4 (1.01–1.65)	1.22 (0.88–1.63)	1.38 (1.07–1.69)	0.193
< 800	13/103 (12.6)	7/33 (21.2)	6/70 (8.6)	0.071
Missing	3	1	2	–
Hb, g/dl	13.2 ± 2.1	12.9 ± 2.2	13.3 ± 2.0	0.372
ASAT, UI/l	52.0 (28.8–90.3)	68.0 (39.5–95.0)	50.5 (26.3–86.0)	0.124
ALAT, UI/l	35.5 (24.8–68.3)	32.5 (24.8–62.8)	39.5 (23.5–73.0)	0.712
ALAT > 40 UI	48 (45.3)	13 (38.2)	35 (48.6)	0.316
Total Bilirubin, µmol/l	8.0 (6.2–12.0)	10.4 (6.2–14.2)	7.7 (5.9–10.7)	0.123
Direct Bilirubin, µmol/l	4.3 (3.0–6.1)	5.7 (3.2–7.7)	3.8 (2.9–5.8)	0.059
Pro BNP, pg/ml	119.5 (45.0–633.8)	279.5 (47.3–1355.3)	98.0 (38.3–378.3)	0.028
Ferritin, ng/ml	1200.0 (565.4–1200.0)	1200.0 (842.5–1200.0)	1200.0 (527.1–1200.0)	0.377
Na^+^, mmol/l	137.9 ± 4.4	137.6 ± 5.8	138.1 ± 3.7	0.593
K^+^, mmol/l	3.8 ± 0.5	3.8 ± 0.5	3.8 ± 0.4	0.934
HbA1c, %	8.3 ± 2.7	8.1 ± 2.7	8.4 ± 2.8	0.737
Creatinin, µmol/l	89.5 (74.0–110.3)	100.5 (74.8–125.5)	85.5 (74.0–105.3)	0.164
LDH, UI/l	410.0 (232.0–656.3)	604.0 (244.0–874.8)	362.5 (228.3–551.8)	0.026
> 245	68/98 (69.4)	23/30 (76.7)	45/68(66.2)	0.299
Missing	8	3	5	–
CK, UI/l	199.0 (96.0–398.0)	274.0 (99.5–485.8)	163.0 (93.0–354.0)	0.214
> 185	46/91 (50.5)	18/28 (64.3)	28/63 (44.4)	0.081
Missing	15	5	10	–
TC, mmol/l	4.2 (3.3–5.6)	4.2 (3.3–5.7)	4.3 (3.2–5.5)	0.927
HDLc, mmol/l	0.93 (0.67–1.24)	1.19 (0.75–1.55)	0.85 (0.66–1.08)	0.047
Triglycerids, mmol/l	1.40 (0.94–2.19)	1.51 (0.79–2.15)	1.36 (1.09–2.38)	0.430
Troponin, ng/l	9.9 (3.2–27.9)	20.8 (10.3–90.5)	4.9 (2.0–16.9)	< 0.0001
> 28	24 (24.5)	14 (41.2)	10 (15.6)	0.005
PTr, %	74.3 ± 16.6	75.4 ± 17.0	73.8 ± 16.6	0.674
< 70	35/93 (37.6)	11/28 (39.3)	24/65 (36.9)	0.829
≥ 70	58/93 (62.4)	17/28 (60.7)	41/65 (63.1)	–
Missing	13	3	10	–
D-dimer, ng/ml	1603.5 (795.3–4093.3)	1694.2 (921.6–5482.1)	1593.3 (744.5–3329.7)	0.430
≤ 500	14/97 (14.4)	5/31 (16.1)	9/66 (13.6)	0.305
> 500–≤ 1000	18/97 (18.6)	3/31 (9.7)	15/66 (22.7)	–
> 1000	65/97 (67.0)	23/31 (74.2)	42/66 (63.6)	–
Missing	9	2	7	–
Fibrinogen, g/l	7.3 (5.4–8.4)	7.4 (6.3–9.1)	7.2 (5.2–8.2)	0.254
CRP, mg/l	125.0 (53.0–218.0)	209.5 (107.0–309.3)	95.5 (29.5–187.8)	< 0.0001
PCT, ng/ml	0.200 (0.095–0.620)	0.360 (0.185–2.503)	0.140 (0.060–0.440)	< 0.0001
< 0.1	26 (24.8)	1 (2.9)	25 (35.2)	0.002
0.1–< 0.25	30 (28.6)	11 (32.4)	19 (26.8)	
0.25–< 0.5	18 (17.1)	6 (17.6)	12 (16.9)	
≥ 0.5	31 (29.5)	16 (47.1)	15 (21.1)	
PaO2/FiO2	100.2 (63.2–209.4)	67.6 (57.9–96.5)	145.5 (73.1–251.2)	0.001
TDM Score				
Normal	3 /92(3.3)	0/27(0.0)	3/65 (4.6)	< 0.0001
Score 1	11/92 (12.0)	0/27(0.0)	11/65(16.9)	
Score 2	17/92 (18.5)	3/27 (11.1)	14 /65(21.5)	
Score 3	27/92 (29.3)	4/27 (14.8)	23/65 (35.4)	
Score 4	22/92 (23.9)	11/27 (40.7)	11 /65(16.9)	
Score 5	12/92 (13.0)	9/27 (33.3)	3/65 (4.6)	
Missing	14	5	9	

Data are mean ± standard, median (IQR), n (%), or n/N (%). Percentage are based on the total number of non-missing values in each category and not necessarily on the total number of participants. P values were calculated by Mann–Whitney U test, χ^2^ test, or Fisher’s exact test, as appropriate. *ALAT* Alanina Amino Transferase, *ASAT* aspartate amino transferase, *CRP* C reactive protein, *Hb* haemoglobin, *HDLc* high density Lipoprotein cholesterol, *LDH* lactate dehydrogenase, *CK* creatinine kinase, *Pro BNP* brain natriuretic peptide, *PTR* prothrombine time ratio, *PCT* Procalcitonin, *TC* total cholesterol, *WBC* white blood cell

**Table 3:** Patients management and evolution

Variable	Total (n = 106)	Non-survivors (n = 34)	Survivors (n = 72)	p-value
Oxygen therapy at admission				
Ambient air	10 (9.5)	0 (0)	10 (14.1)	< 0.0001
Nasal cannula oxygenotherapy	18 (17.1)	2 (5.9)	16 (22.5)	
High concentration oxgen masks	68 (64.8)	24 (70.6)	44 (62.0)	
Non invasive ventilation	9 (8.6)	8 (23.5)	1 (1.4)	
ARDS severity				
No ARDS	7/66 (10.6)	0/24 (0.0)	7/42 (16.7)	0.003
Mild ARDS	11/66 (16.7)	2/24 (8.3)	9/42 (21.4)	
Moderate ARDS	15/66 (22.7)	3/24 (12.5)	12/42 (28.6)	
Severe ARDS	33/66 (50.0)	19/24 (79.2)	14/42 (33.3)	
Missing	40	16	24	
AKI	15/74 (20.3)	12/30 (40.0)	3/44 (6.8)	< 0.0001
Missing	32	14	18	
Hemodialysis	14 (13.2)	13 (38.2)	1 (1.4)	< 0.0001
ARDS	59/66 (89.4)	24/24 (100)	35/42 (83.3)	0.034
Missing	40	16	24	
Mechanical ventilation	28 (26.4)	28 (82.3)	0 (0.0)	–
Vasopressors use	9 (10)	9 (32.1)	0 (0.0)	–
Delay from symptoms onset to				
Corticosteroids start, day	10.0 (7.0–15.0)	11.0 (8.0–13.0)	10.0 (6.0–15.0)	0.495
NIV initiation, day	12.0 (7.0–13.0)	12.0 (7.0–13.0)	–	–
Mechanical Ventilation initiation, day	15.0 (9.0–22.0)	15.0 (9.0–22.0)	–	–
Death or discharge, day	19.0 (15.0–26.0)	22.0 (14.0–33.0)	18.0 (15.0–22.0)	0.113

Data are median (IQR), n (%), or n/N (%). Percentage are based on the total number of non-missing values in each category and not necessarily on the total number of participants. P values were calculated by Mann–Whitney U test, χ^2^ test, or Fisher’s exact test, as appropriate. *AKI* acute kidney injury, *ARDS* acute respiratory distress syndrome, *NIV* non-invasive ventilation

**Table 4:** Predictors of mortality in COVID-19 patients

Variable	Unadjusted HR (95% CI)	p	Adjusted HR (95% CI)	p
Age*	1.05 (1.02–1.09)	**0.005**	1.38 (1.10–1.82)	**0.018**
Proteinuria				
Negative	1		1	
Positive	3.72 (1.27–10.94)	**0.017**	2.60 (1.40–6.42)	**0.009**
RR*	1.06 (1.02–1.11)	**0.008**	1.42 (1.09–1.92)	**0.019**
Troponin*	1.03 (10.1–10.5)	**0.001**	1.79 (0.35–9.10)	0.481
CRP*	1.04 (1.01–1.07)	**0.007**	1.03 (0.99–1.07)	0.288
AKI				
No AKI	1		1	
AKI 2	2.61 (0.86–7.97)	0.091	0.91 (0.26–3.26)	0.890
AKI 3	4.47 (1.59–12.58)	**0.005**	2.51 (1.33–6.80)	**0.016**
PCT*	1.09 (1.04–1.14)	< 0.001	1.08 (1.03–1.14)	**0.002**
ProBNP*	1.09 (0.75–1.86)	0.285	1.19 (0.20–1.36)	0.288
LDH*	1.09 (0.99–1.19)	0.077	1.50 (0.35–1.68)	0.633
PaO2/FiO2*	1.43 (0.99–1.99)	0.636	1.10 (0.98–1.98)	0.284
SBP*	1.03 (0.02–1.28)	0.722	1.54 (0.39–1.69)	0.101
Neutrophils*	1.03 (0.20–1.43)	0.070	1.00 (0.301.61)	0.866

Bold values are the statistically significant p defining the variables (factors) associated to mortality

*AKI* acute kidney injury, *RR* respiratory rate, *CRP* C-reactive protein, *PCT* Procalcitonin, *SBP* systolic blood pressure, *HR* hazard ratio *variables continues

## Discussion

To date, only two studies have been published from the DRC examining patients admitted with COVID-19 [17, 18]. Both these studies were limited by a lack of robust analysis of biological and laboratory parameters that might predict hospital mortality in COVID-19 [7, 18]. Our retrospective cohort study, carried out in the DRC, aims to add comprehensive data about mortality risk factors for COVID-19. The findings demonstrate that age, respiratory rate, proteinuria, procalcitonin and AKI were significantly associated with mortality in COVID-19 patients. Additionally, increasing levels of creatinine during hospital admission were associated with an increased mortality.

Globally, the hospital COVID-19 mortality rates varies between 4 and 70% [19–25]. This disparity is partially explained by differences in the epidemiology of the study populations as well as in their hospital management. For example, Du et al. demonstrated that older patients with pre-existent co-morbidities had a higher risk of mortality than a younger healthier person [25]. Ciceri et al. reported 23% mortality in patients presenting less severe forms on admission (a median oxygen saturation of 93%) [26]. In comparison, our study revealed a mortality of 32.0% of whom only 24.5% had an age > 65 years, and had few comorbidities and upon admission had a less severe form of the disease (mean PaO2 62.62 ± 14.0 mmHg). In contrast to international studies demonstrating that being male is associated with an increased risk of mortality [27], our findings did not demonstrate any significant gender difference in risk. All intubated patients died, reflecting the difficulty to manage mechanical ventilation of COVID-19 patients during the first wave in the context of lack of experience and clear recommendations.

As in several previous studies [25–29], in our study an advanced age was associated with increased mortality from COVID-19. This vulnerability amongst the elderly is often explained by immunoscenescence that is accompanied by a decrease in the production of native T and B cells as well as a decrease in the function of immune cells participating in innate immunity [28]. These changes reduce the effective viral clearance and increase the likelihood of triggering a deregulated immune response in which cytokines are largely released from activated immune cells causing a cytokine storm [28]. In addition to immune senescence, there are several other age-related factors such as comorbidities resulting in higher morbidity and mortality [28]. In our cohort, the number of comorbidities also increased with age.

Viral infections are not usually associated with a raised serum PCT, a finding supported in current COVID-19 research [30]. Procalcitonin, which is the 116-amino acid precursor of the hormone calcitonin, is normally synthetized and released by thyroid parafollicular C cells [30]. It can also be synthetized in many extrathyroid tissues during bacterial infection which is mediated by increased concentration of tumor necrosis factor alpha (TNFα) and interleukin 6 [30]. Worldwide, the average PCT level on admission is less than 0.25 μg/L in COVID-19 patients [31]. During admission for COVID-19, an increased PCT is explained either by a bacterial hospital acquired co-infection or by a general deterioration of the patient [32]. Several studies have reported that elevated PCT is positively associated with the severity of COVID-19 [10, 33–35]. Hu et al. describe bacterial co-infection rates, defined by a positive blood culture in 20% of those who were severely unwell and in 50% who were critically unwell. Yet, in 50% of those with severe COVID-19 and in 80% of those critically unwell the PCT was raised [30]. In our study, 12/81 (14.8%) of admission blood cultures were positive yet the PCT was raised in 29.5% of those patients. Our study demonstrated that during infection with COVID-19 a progressive elevation of PCT served as a marker for a poor prognosis. This finding was supported by a study by Lippi et al. [36]. Unlike the increase in creatinine, procalcitonin decreases over time in non-survivors and can suggest that the worse outcome of COVID-19 patients in the study may be secondary to organ dysfunction and not superinfection.

Acute Kidney Injury (AKI) affects approximatively 20–40% of COVID-19 patients admitted to intensive care [37]. It is considered as a marker of disease severity and a negative prognostic factor for survival [37, 38]. AKI can lead to impaired acid–base, fluid, and electrolyte homeostasis, all of which may contribute to worse outcomes for patients with COVID-19 [38]. In our cohort the incidence of AKI was 16.2%. A progressive elevation of creatinine was noted as a marker for poor prognosis, yet, only AKI stage 3 was found to be an independent risk factor associated with mortality. AKI is a well-recognised factor of poor prognosis but during the SARS Cov-2 pandemic few studies have found a significant association between AKI and death [37]. This might be explained by the findings of Cheng et al. who demonstrated that only AKI Stages 2 or 3 are associated with a high risk of mortality [39]. Proteinuria is not only a marker for kidney disease or its progression, but also a manifestation of systemic disease in the kidney. Although transient, proteinuria is reported to be a disease severity marker and a mortality risk cause in Intensive care unit (ICU). As in previous studies [40, 41], proteinuria was associated with increased mortality in ICU COVID-19 patients.

### Strengths and limitations

One of the principle weaknesses of this study is that it was carried out in a single centre thus the results cannot be generalised to all COVID-19 patients. Another weakness is that because it is retrospective we were unable to obtain all data related to the parameters of interest. This being a private hospital localized in the COVID-19 epicentre area and providing specialized tertiary care, can therefore likely to represent a selected group and lead to an overestimation of COVID-19 mortality. Finally, the small sample size was not sufficiently powered to identify potential associations between variables of interest. Nevertheless, this study has the advantage of being the first one in the DRC to examine epidemiological and laboratory data during the course of the admission to evaluate some of the risks factors associated with mortality among COVID-19 patients.

## Conclusion

Mortality rate of COVID-19 patients is high, particularly in intubated patients and is associated with age, respiratory rate, procalcitonin, proteinuria and acute kidney injury.

## Data Availability

The datasets used and/or analysed during the current study are available from the corresponding author on reasonable request.

## Abbreviations

AKI: Acute kidney injury
ARDS: Acute respiratory distress syndrome ARDS
BOS: Blood oxygen saturation
CK: Creatinine kinase
CKD: Chronic kidney disease
CRP: C-reactive protein
CT: Computer tomography
Covid-19: Coronavirus disease 2019
DRC: Democratic Republic of Congo
FiO2: Inspired oxygen fraction
HB: Hemoglobin
HR: Heart rate
KMC: Kinshasa medical center
LDH: Lactate dehydrogenase
MV: Mechanical ventilation
PaO2: Arterial oxygen pressure
PCT: Procalcitonin
PTr: Prothrombin rate
RR: Respiratory rate
RT-PCR: Reverse transcriptase polymerase chain reaction
SARS-Cov-2: 2019 Novel coronavirus
WBC: White blood cell
WHO: World Health Organization
